# Access experiences and attitudes toward abortion among youth experiencing homelessness in the United States: A systematic review

**DOI:** 10.1371/journal.pone.0252434

**Published:** 2021-07-01

**Authors:** Sarah Munro, Savvy Benipal, Aleyah Williams, Kate Wahl, Logan Trenaman, Stephanie Begun

**Affiliations:** 1 Department of Obstetrics & Gynaecology, Faculty of Medicine, University of British Columbia, Vancouver, BC, Canada; 2 Centre for Health Evaluation and Outcome Sciences, Providence Health Care Research Institute, Vancouver, BC, Canada; 3 Royal College of Surgeons in Ireland, Dublin, Ireland; 4 Faculty of Pharmaceutical Sciences, University of British Columbia, Vancouver BC, Canada; 5 Factor-Inwentash Faculty of Social Work, University of Toronto, Toronto, ON, Canada; Center for Primary Care and Public Health, SWITZERLAND

## Abstract

**Objectives:**

We sought to review the literature on the access experiences and attitudes toward abortion among youth experiencing homelessness in the United States.

**Methods:**

We conducted a systematic review of peer‐reviewed literature published from 2001 to 2019. We included qualitative studies involving US participants that focused on access experiences, views, or accounts of unintended pregnancy and/or abortion among youth experiencing homelessness. We excluded studies published before 2001 as that was the year mifepristone medication abortion was made available in the US and we aimed to investigate experiences of access to both medical and surgical abortion options.

**Results:**

Our thematic analysis of the data resulted in five key themes that characterize the abortion attitudes and access experiences of youth experiencing homelessness: (1) *engaging in survival sex and forced sex*, (2) *balancing relationships and autonomy*, (3) *availability does not equal access*, (4) *attempting self-induced abortions using harmful methods*, and (5) *feeling resilient despite traumatic unplanned pregnancy experiences*.

**Conclusions:**

Youth experiencing homelessness experience barriers to abortion access across the US, including in states with a supportive policy context and publicly funded abortion services. In the absence of accessible services, youth may consider harmful methods of self-induced abortion. Improved services should be designed to offer low-barrier abortion care with the qualities that youth identified as important to them, including privacy and autonomy.

## Introduction

Each year in the United States, approximately 3.5 million (1 in 10) youth aged 18 to 25 experience homelessness [1, 2]. While pregnancy rates have declined over time among youth in the US, the limited data available suggest that pregnancy rates in this population are higher than those among housed youth [2], and as many as half of women experiencing homelessness report a previous pregnancy [3–5]. Over the past 20 years, adolescent girls have continued to have 1 in 2 odds of becoming pregnant while experiencing homelessness [3, 5]. In the large national study, *Missed Opportunities*: *Pregnant and Parenting Youth Experiencing Homelessness in America*, it was estimated that 10% of homeless and underhoused female youth aged 13 to 17 and 44% of those aged 18 to 25 experience pregnancy or parenthood [1]. In comparison to their housed peers, youth experiencing homelessness are at greater risk of becoming pregnant, and have higher rates of miscarriage and lower rates of abortion [4, 6]. Youth experiencing homelessness also are less likely to access reproductive health care services due to health system factors, such as cost of care, restrictive legislation, and insurance coverage [7, 8].

Adolescents in the US have had the choice between medication or surgical options for first trimester induced abortion since 2000, when the drug mifepristone was approved for this purpose. People seeking to terminate pregnancy strongly value having a choice between medication and surgical abortion [9, 10] and receiving information to support their informed decision-making, regardless of their age or housing situation [11, 12]. There is evidence, however, that clinicians may be hesitant to offer the option of medication abortion to marginalized populations. This hesitation may arise from a confluence of factors, including lack of provider knowledge about the option of medication; provider perceptions that patients are disinterested in this option; and provider concerns about feasibility related to patients using the medication correctly, managing the abortion in an unstable living situation, and loss to follow up [13, 14]. Simultaneously, there is a small but alarming body of evidence that youth, including youth experiencing homelessness, may opt for self-induced abortion using harmful methods rather than seek surgical abortion care [6, 8, 15–17]. These studies signal the need for improved decision support so that youth can make informed, evidence-based decisions about safe method of abortion. From a reproductive justice standpoint [18], youth experiencing homelessness have the right to maintain personal bodily autonomy, including the choice of method of abortion, like their housed peers. Method of first trimester induced abortion is a preference-sensitive decision where there are multiple options, each with trade-offs for risks and benefits to the patient, and thus the choice depends on the patient’s preferences and individual needs [19]. In order to support informed choices, it is first necessary to understand *what* informs decisions about safe method of abortion, from the perspectives of youth experiencing homelessness themselves, so that supports can be patient-oriented and responsive to youth experiences and attitudes.

Although pregnancy among youth experiencing homelessness is relatively common and it is important to support reproductive choice for this vulnerable population, there are few examples of research investigating their attitudes towards pregnancy and experiences seeking abortion care. The purpose of this narrative review is to fill this knowledge gap by synthesizing qualitative research on the access experiences and attitudes toward abortion among youth experiencing homelessness in the US.

## Study design

We conducted systematic searches in February 2020 in CINAHL, Medline, and Embase databases. We chose these databases for their reliability in identifying peer-reviewed qualitative research. A full search strategy including MESH terms, designed in collaboration with a medical subject librarian (E.S-W.), can be found in S1 Appendix. We used PICo elements for qualitative studies to frame our search strategy, where the population (P) was youth experiencing homelessness, the phenomenon of interest (I) was attitudes and experiences, and the context (Co) was unintended pregnancy and/or accessing abortion. We report the results of our synthesis following the Enhancing Transparency in Reporting the Synthesis of Qualitative Research (ENTREQ) guidelines [20].

### Study selection

Studies eligible for inclusion were peer-reviewed, in English, published after 2001, involved US participants, and had full text available; focused on experiences, views, or accounts of unintended pregnancy and/or abortion among youth experiencing homelessness; and used qualitative study designs. We chose to limit our results to those published after 2001 with participants from the US for two reasons. One, medication abortion with mifepristone received approval from the US Federal Drug Agency in September 2000, and we wanted to capture studies after 2001 where participants may have had access to both mifepristone and surgical abortion options. Two, we limited our search to studies from the US because of calls from organizations for US-specific evidence to inform improvement of sexual and reproductive health programs for youth experiencing homelessness in the US [21, 22]. Any definition of “youth” was accepted due to the disparity in the term and the scarcity of information regarding this topic.

For our purposes, youth experiencing homelessness are defined as individuals in the developmental stage of emerging adulthood who lack a fixed, regular, and adequate nighttime residence [23]. This is similar to the broadest US federal definition of youth homelessness, which includes any “individual who is less than 21 years of age, for whom it is not possible to live in a safe environment with a relative, and who has no other safe alternative living arrangement” (42 U.S.C. § 5732). Living arrangements for these youth can include living temporarily in hostels or shelters, couch surfing with friends, renting inexpensive rooms in boarding house or hotels, or in some cases, sleeping on the streets. They may also live with a relative or friend who is at imminent risk of losing their housing. Youth experiencing homelessness are characterized in part by the *instability* of their housing.

### Study screening, and appraisal

Using the search terms and strategy provided in S1 Appendix, our initial search identified 314 studies. One trainee author (SB) screened results for duplicates, removing 38 studies, leaving 276 articles which we subjected to a three‐stage process for study selection: title, abstract, and full‐text review. At each stage, two independent reviewers (SB, SM) assessed the citations against inclusion criteria. Differences in assessment were resolved by consensus. We also hand-searched the reference lists of articles and sought input from subject expert for additional studies that might meet our inclusion criteria. We used the Critical Appraisal Skills Programme (CASP) Qualitative Research Checklist [24] to assess the quality of included studies in the following domains: research aims, design, methodology, recruitment, data collection, data analysis, ethical issues, participant-researcher relationship, findings, and research value. High quality studies met criteria of 8 or more items on the CASP Qualitative Research Checklist, medium quality studies met that of 5–7 items, and low-quality studies met that of less than 5 items. Two authors (AW, SM) independently evaluated the quality of each included publication and resolved disagreements through discussion until consensus was reached.

### Charting, collating, summarizing and reporting the results

We developed a data extraction template designed to collect comparable data on study characteristics and results. We pilot‐tested the form on two randomly‐selected articles by two independent reviewers (SB, SM) and refined as needed. We extracted information about the sample characteristics (size, age range, geographic location), study design (methods), and results.

To appraise and synthesize key concepts, we conducted a thematic analysis following Braun and Clarke’s reflexive thematic analysis techniques [25–27]. First, two researchers (SB, SM) immersed ourselves in the literature, reading and re-reading each study while coding the results of included studies using open and *in vivo* codes. Results data included participant quotations and authors’ interpretations included in the text, data tables, and S1 & S2 Appendices. Next, we collated and collapsed codes with similar meaning into an initial coding framework. Then, one author (SB) used this coding framework to code the results in their entirety, keeping memos and an audit trail to their document analytic choices. We met regularly to discuss the coding and to identify synergies, discrepancies, and patterns within and between the studies.

## Results

**Fig 1:**
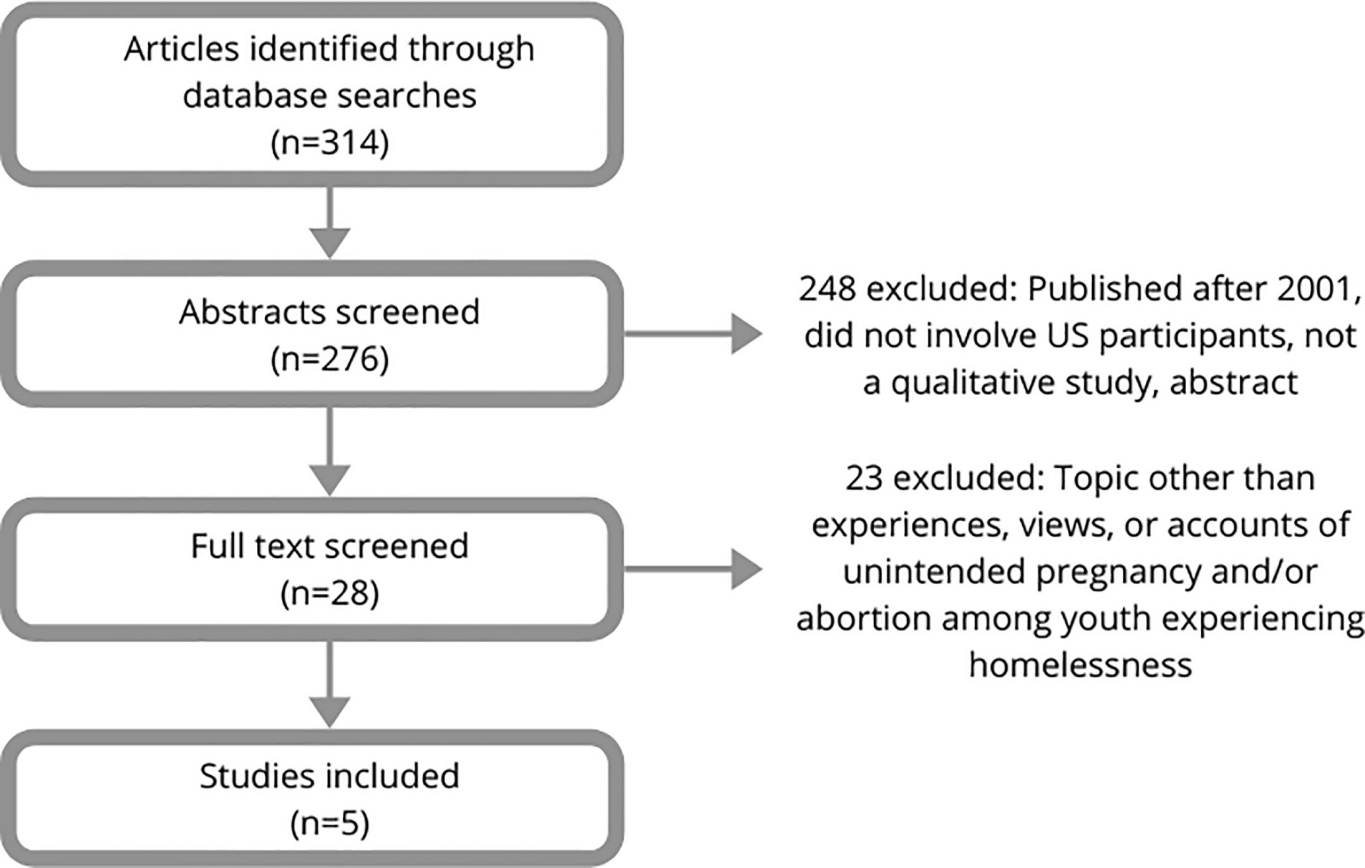
Selection of included studies.

Our systematic search yielded five qualitative research studies eligible for analysis (Fig 1) [6, 15–17, 28]. As described in Table 1, studies included 132 participants from California (n = 2), Colorado (n = 1), Texas (n = 1), and Washington State (n = 1), were published between 2001 and 2019, and included youth as young as 15. Quality appraisal using the CASP checklist identified that included studies were determined to be of high [6, 17, 28] and medium [15, 16] quality (Table 2). We used classification data from the Guttmacher Institute to determine how ‘hostile’ or ‘supportive’ states’ policy environments were toward abortion at the time of each study’s publication [29]. Abortion policies in California were ‘supportive,’ in Washington State were ‘leans supportive,’ in Colorado were ‘middle- ground,’ and in Texas they were ‘hostile.’

**Table 1:** Characteristics of included studies.

Author & Date	Title	Sample Characteristics	Setting	Topic	Study Design
Begun et al (2018)	‘I Know They Would Kill Me”: Abortion Attitudes and Experiences Among Youth Experiencing Homelessness.	N = 30	Denver, Colorado	Youth knowledge and experiences with access to abortion	Semi-structured interviews
		Age: 18–21			
Cronley, Hohn & Nahar (2018)	Reproductive health rights and survival: The voices of mothers experiencing homelessness	N = 20	Texas	Reproductive health histories of women currently experiencing homelessness (e.g. What were your feelings when you discovered that you were pregnant?)	Semi-structured interviews
		Age: 38 (average)			
Smid, Bourgois & Auerswald (2011)	The challenge of pregnancy among homeless youth: reclaiming a lost opportunity	N = 21	Berkeley, California	Experiences of pregnancies among street youth, including pregnancy and termination outcomes and experiences	Semi-structured interviews and participant observation
		Age: 18–26			
Hathazi et al (2009)	Pregnancy and sexual health among homeless young injection drug users.	N = 41	Los Angeles, California	Pregnancy and sexual health experiences, including miscarriages and terminations, among homeless youth with a history of IV drug use	Semi-structured interviews
		Age: 16–29			
Ensign (2001)	Reproductive Health of Homeless Adolescent Women in Seattle, Washington, USA	N = 20	Seattle, Washington	Homeless adolescent females’ experiences of health issues, self-care, and fertility control (pregnancy, birth control, and abortion)	Semi-structured interviews and focus group discussions
		Age: 15–23			

**Table 2:** Quality appraisal of included studies.

	Begun et al (2018)	Cronley, Hohn & Nahar (2018)	Smid, Bourgois & Auerswald (2011)	Hathazi et al (2009)	Ensign (2001)
Clear statement of the aims of the research?	Yes	Yes	Yes	Yes	Yes
Was a qualitative methodology appropriate?	Yes	Yes	Yes	Yes	Yes
Was the research design appropriate for the aims?	Yes	Yes	Somewhat	Somewhat	Yes
Was the recruitment strategy appropriate for the aims?	Yes	No	Somewhat	Somewhat	Yes
Were the data collected in a way that addressed the research issue?	Yes	Somewhat	Yes	Somewhat	Yes
Has the relationship between researcher and participants been adequately considered?	No	No	Somewhat	No	No
Have ethical issues been taken into consideration?	Yes	Yes	Yes	Yes	Yes
Was data analysis sufficiently rigorous?	Yes	Yes	Yes	Cannot tell	Yes
Is there a clear statement of findings?	Yes	Yes	Yes	Yes	Yes
How valuable is the research?	High	Medium	High	Low-Medium	High

Our thematic analysis of the data resulted in five key themes that characterize the abortion attitudes and experiences of youth experiencing homelessness: (1) *engaging in survival sex and forced sex*, (2) *balancing relationships and autonomy*, (3) *availability does not equal access*, (4) *attempting self-induced abortions using harmful methods*, and (5) *feeling resilient despite traumatic unplanned pregnancy experiences*.

### Engaging in survival sex and forced sex

Youth experiencing homelessness reported that they or someone they knew were involved with survival sex–engaging in sexual intercourse in exchange for food, drugs, housing, or money. For some participants, unexpected pregnancy co-occurred with survival sex, addiction, and abuse [15]. In most instances of an unplanned pregnancy resulting from survival sex or forced sex, participants described delaying seeking prenatal care, seeking abortion, or attempting self-induced abortions using harmful methods [6, 15, 28]. One participant described choosing a self-induced abortion using harmful methods after forced sex, saying *“‘cause I was raped and I didn’t want to be pregnant*. *I was thirteen*. *So I got somebody I know who’d kick my ass–so I did and I wasn’t pregnant anymore”* [28]. For many, survival sex was related to previous experiences of sexual assault and forced sex as young girls, as one participant stated, “*It’s most common for homeless women I’ve known to have been raped both before and after hitting the streets*” [28]. Participants emphasized that survival sex also was a strategy to acquire basic needs [15]. Some female youth, for instance reported feeling vulnerable on the street and fearing for their safety [28]. In the study by Ensign [28], female participants sympathized with their peers engaging in survival sex, with one participant stating: “*I think they just want to feel loved for a few moments*, *you know*, *and feel like someone actually does care about ‘em*.*”* When describing how survival sex and forced sex impacted their access to sexual and reproductive health services, participants emphasized that it is difficult to discuss and requires a health care provider to build trust and use non-shaming language. Another participant from Ensign et al.’s study reflected, “*I think I’ve looked down at some of my friends for having slept with guys*, *to stay at their places*. *And basically*, *if your best friends have that much stigma about it*, *trusting a health care provider is going to be that much harder*” [28]. These experiences provide insight into the context of choices and what informs decisions to seek or avoid health services.

### Balancing relationships and autonomy

Participants who experienced pregnancy described increased stress and tension regarding how to manage an unplanned pregnancy. Decisions to continue or terminate pregnancy created real or anticipated strain on relationships with their family or their partner [6, 17, 28]. As one young woman articulated, she was reluctant to consider abortion because of fear of retaliation from her parents: “*My parents are super against abortion so I know they would kill me*, *like literally kill me and end my life*, *if they ever found out about me having an abortion*, *so I think that’s sort of shaped my view*, *in just that I don’t think I could*. *Not because I think it’s really wrong or anything but mostly because I wouldn’t want to die myself*” [17]. Some respondents stated that homelessness was a direct cause of obtaining abortion without familial consent. Most youth expressed that their families had strong anti-abortion views and would “*banish [them] for life*” if abortion were to be obtained [17]. For those youth under the age of 18 who were experiencing homelessness, they were not able to have an abortion without parental consent, further illuminating the limits to their autonomy in decision-making [28].

Some youth in long-term relationships described the decision-making process as a collaborative negotiation where the pregnant person had the final say, while others disagreed with their partner about whether or not to terminate a pregnancy. Most youth had unstable housing, moved frequently, or feared their heavy substance use would have a detrimental effect on their ability to parent a child. As one youth described, “*So it’s obvious that we’re not going to be the perfectly compatible couple*. *[When we were making the decision about whether to become parents] that was the only time in our relationship that violence between us was involved*” [6]. Another participant noted that she terminated her pregnancy to “*keep [her partner] happy*” [17].

### Availability does not equal access

Most participants experiencing homelessness resided in states that had relatively supportive abortion policies and low- or no-cost abortion services. However, the overwhelming majority of youth lacked awareness of and correct information about abortion services. For instance, adolescents in Washington State did not want healthcare authorities to find out “*they were run-aways*” [28], as they misperceived the state would notify their parents or require parental consent for an abortion. They also may not have been familiar with options for ‘judicial bypass’–an order from a judge allowing a minor to have an abortion without notifying anyone. Although parental notification is not required in the state, one young participant described it is typical to avoid health services and attempt self-induced abortion using harmful methods: “*If you’re pregnant and you’re on the streets*, *you don’t tell anyone because they’ll tell someone else and it’ll get around eventually to the state*. *So you don’t tell anyone*, *you do the abortion yourself*, *and it’s like–‘I was never pregnant*’” [28]. Other participants sought to avoid abortion services because of past negative experiences with reproductive health care providers. For instance, one participant’s reproductive rights were violated after a traumatic birth experience where she felt coerced into tubal ligation [15].

The cost of abortion care also was a perceived access barrier for most youth, who expressed inflated perceptions of abortion costs: “*abortions cost like a couple thousand dollars*” [17]. These barriers were not consistent with studies conducted in California [6, 16], where youth were largely aware of and able to access subsidized reproductive care services. In a study involving 41 youth experiencing homelessness in Los Angeles, 80% of participants in confirmed their pregnancy at a healthcare clinic and continued to receive some form of care [16]. In contrast, one participant who had four abortions in California described how traveling companions from other states had resorted to using ‘*home remedies’* where subsidized abortion services were less accessible [6].

### Attempting self-induced abortions using harmful methods

None of the included studies provided insights into experiences of and attitudes toward medication and surgical abortion in formal healthcare settings. Rather, youth in the included studies described examples of a harmful third approach outside the medical system, ‘attempted self-induced’ abortion: attempting to induce a miscarriage through heavy use of substances or other non-evidence based, harmful methods. Attempted self-induced abortion was distinct from and did not include evidence-based ‘self-managed’ abortion strategies that offer promising support for marginalized people, such as on-line or telemedicine services to assist self-sourced mifepristone. Youth experiencing homelessness reported seeking or attempting self-induced abortions regardless of whether their state’s abortion policies were “hostile,” or “supportive” [30]. In geographic regions where at the time of the study publication abortions were more difficult to obtain, youth did perceive that there was a lack of accessible abortion services, which may have been related to regional access [15, 17]. Notably, youth in more supportive policy climates also reported decreased use of sexual and reproductive services and experiences of self-induced abortion for reasons other than geographic access, such as embarrassment and stigma [6, 16, 28]. In the qualitative study involving 20 young women experiencing homelessness in Washington State, 16 indicated they knew at least one other woman who had attempted self-induced abortion and 4 reported having attempted a self-induced abortion themselves [7]. In the study involving 30 youth recruited from a Denver-area shelter, each of the participants either knew of someone who had attempted and/or completed a self-induced abortion, and two reported having “*created a miscarriage*” for themselves to avoid the shame and stigma associated with obtaining an abortion [17].

Attempts at self-induced abortions were stated to be common because youth thought it was easier to “*induce a miscarriage*” rather than “*look like a monster*” for wanting to have an abortion [16, 17]. “*In some cases*,*”* Smid and colleagues wrote, *“young women found their situation so overwhelming they attempted to ignore the pregnancy by increasing their use of drugs and alcohol*” [6]. Methods used for attempting an abortion included: starvation, heavy substance abuse; planned physical abuse by themselves, a friend, or partner (“*beating it out*”) [28]; insertion of sharp objects, coat hangers, or bleach-soaked tampons; and drinking bleach or herbal abortifacients [17]. Some youth were already using drugs prior to conceiving and increased their substance use in order to “*forget about it*” or to get “*so high*, *nothing could survive*” [16, 28]. These pregnant individuals were aware that attempting self-induced abortions could harm them.

### Feeling resilient despite traumatic unplanned pregnancy experiences

Finally, youth experiencing homelessness may also experience resilience and optimism after experiences of unplanned pregnancy [28]. For those who chose to continue their unplanned pregnancies to birth, pregnancy often was a catalyst for personal transformation [6]. Regardless of whether youth chose to continue an unplanned pregnancy or seek termination, the experience of becoming pregnant catalyzed motivation to change behaviour and find shelter: “*We got really sick of doing what we were doing*, *and since I was pregnant we knew we had to make some drastic changes*” [16]. For others, feeling resilient involved believing that their experiences of pregnancy and abortion were an opportunity to “*give back*” to the community who supported them and provided a “*sense of family*” during that time [15, 28]. One described this as using their experiences of reproductive trauma and abortion while homeless to guide other women to safety: “I’m gonna come to you with, ‘Hey, this is what I know because I walked it’” [15].

## Discussion

In this narrative review we identified that youth experiencing homelessness can have unplanned pregnancies resulting from survival sex or forced sex and do not always feel they can make an autonomous choice to have an abortion. They may also consider the potential negative impact of their choice on their relationship with their family or partner. Even in states that had highly supportive abortion policies and no-cost abortion care, youth experiencing homelessness still perceived there were barriers to abortion access. These barriers were mostly based on personal perceptions and included the belief that a ‘miscarriage’ will elicit more sympathy from peers, assumptions that the abortion will be expensive (not publicly funded), and fear that family members may be notified. The results of this review also identified that homeless and underhoused youth perceive it is common for their peers to attempt self-induced abortion using harmful methods. One hopeful theme identified in this review is that, for youth experiencing unplanned pregnancy, the experience can sometimes lead to a feeling of resilience, particularly as a motivation to give back to their community, find housing, or adopt more healthy behaviours. Our search yielded only five qualitative studies on this topic, pointing to the need for further research in this field.

One of the patterns in our data reveals a poorly understood phenomenon about access to abortion care in states with highly available abortion services. Even in states with supportive abortion policy climates and relatively affordable and socially acceptable abortion services, self-induced abortions appear to be an option considered by youth experiencing homelessness. The prevalence of attempted self-induced abortion using unsafe methods in the general population appears to be lower than that among youth experiencing homelessness [31]. These findings suggest that youth experiencing homelessness lack access to accurate information about abortion options and how to access them in a safe, stigma-free manner and that this knowledge gap may contribute to attempts to self-induce abortion with unsafe methods, even in states with supportive policies, like California. Previous accounts of youth deliberately engaging in heavy substance use may explain why as many as 40% of pregnancies in this population are reported to end in miscarriage [4]. Overall, our results suggest that access to abortion care may vary widely within states and that availability and affordability may not translate to *accessibility* for marginalized populations, including youth experiencing homelessness.

In the studies we reviewed, youth experiencing homelessness demonstrated misconceptions about abortion access, costs, and availability. Some youth avoided seeking abortion services, for instance, because of fears that their parents would be notified, or that it would cost “a couple thousand dollars” out of pocket, or because of previous traumatic experiences with reproductive health services. Based on the available data, it is unclear how these misperceptions arose and what informational interventions would be best suited to improve youth knowledge about abortion care. What are the decisional needs of youth experiencing homelessness who are considering abortion? How accessible and appropriate are existing resources and supports for abortion decision-making? It is critical for future health services research to investigate the information and decision-making needs of youth experiencing homelessness.

Our results also highlight the importance of community in shaping abortion attitudes and providing support for youth. Previous studies highlight that youth learned about abortion methods from family, friends, and peers, suggesting that broader social networks need to be targeted to disseminate accurate information, while at the same time addressing issues of stigma [6, 8, 15–17]. Social workers, for instance, engage in health promotion, education, and advocacy work that intersects with unintended pregnancy and abortion. They and other social service providers in community settings are also positioned to provide referrals for individuals with unplanned pregnancy [32]. Youth typically obtain services after direction or guidance from adults, such as teachers and caseworkers, and these adults act as a ‘gateway’ to services [33]. Sexual and reproductive health care referrals are already provided in low-barrier community settings, such as drop-in centres frequented by youth experiencing homelessness, and these experts are ideally positioned to collaborate with youth to design decision support interventions [34]. Evidence-based strategies that can be incorporated in such settings are decision coaches paired with patient decision aids, which provide information, help clarify youth preferences, and prepare them to make a decision [19, 35]. Decision coaching used alongside patient decision aids is “non-directive support by a trained healthcare provider to help patients/persons prepare for making a health decision” [35]. This model aims to establishing rapport between the coach and patient (youth) and support them in communicating their values and preferences. In trusted, low-barrier settings, decision coaches may also identify and mitigate factors that may make it hard for the youth to implement the chosen option, such as the youth’s relationship with their family or partner, misperceptions about cost barriers to access, and/or their desire to frame pregnancy termination as a miscarriage.

Our review data also did not identify any differences in experiences of and attitudes toward medication and surgical abortion. We specifically designed the review to include only studies conducted after mifepristone medication abortion was made available in the US. As abortion is a preference-sensitive decision involving two options–surgical and medical–we anticipated that included studies would explore experiences of and attitudes toward abortion *method* among youth experiencing homelessness in the US. The included studies did not present data on method selection in formal healthcare settings, rather participants’ experiences involved a troubling third option–self-managed abortion involving harmful techniques outside the formal medical system. Mifepristone medication abortion can be accessed through a traditional, clinic-based model of care, as well as through evidence-based ‘self-managed’ abortion strategies, including on-line or telemedicine services to assist self-sourced mifepristone [36]. Given how youth valued having an abortion that appeared like a ‘miscarriage’ to reduce shame and enhance privacy, it would be useful for future research to investigate the feasibility of medication abortion broadly, and safe self-managed abortion in particular, among youth experiencing homelessness.

### Strengths and limitations

Studies included in this review included participants that were accessing homeless shelters or other medical services. The opinions of these youth may not be adaptable to all youth experiencing homelessness, such as those who are not accessing services or living in more rural areas. None of the studies presented data or interview questions on abortion method selection in formal healthcare settings. This topic may have been outside the scope of the included studies or the authors chose not to report on that aspect of youth decision-making. Social desirability bias and the sensitive topics of sex, unplanned pregnancy, and abortion may have led some youth to under-report their experiences. In a few studies, interviews were completed with both partners present or in small groups. In these cases, youth may have limited what they disclosed to avoid confrontation with partners known to have differing opinions on abortion. We also note that our search yielded a relatively small sample of five studies from participants residing in four US states. Results of this review may not be adaptable for other regions in the US, particularly given the variation in state abortion laws. The experiences of youth in other settings may provide unique, rich insights, and future research could explore the experiences of youth experiencing homelessness in low- and middle-income settings where housing insecurity and access to sexual and reproductive healthcare are significant issues. Despite the small sample of studies, our analysis provides an in-depth understanding of key recurrent themes in the literature and contextual detail that enhances the evidence on abortion access among youth experiencing homelessness in the US. Finally, we built credibility and rigour into the research process by bringing multiple perspectives to the review, noting disagreements in our analysis, and making analytic choices through discussion and consensus.

## Conclusion

Youth experiencing homelessness face barriers to abortion access across the US, including in states with a supportive policy context and publicly funded abortion services. In the absence of accessible services, youth may consider harmful methods of self-induced abortion. Improved services should be designed to offer low-barrier abortion care with the qualities that youth identified as important to them, including privacy and autonomy. Our review is the first synthesis of the qualitative literature on abortion experiences of youth experiencing homelessness in the US.

## Supporting information

S1 Appendix Database search strategy. (DOCX)

S2 Appendix (DOCX)
