# Supplementary material for: Access experiences and attitudes toward abortion among youth experiencing homelessness in the United States: A systematic review
Source: PLoS One. 2021 Jul 1;16(7):e0252434. doi: 10.1371/journal.pone.0252434 (PMC8248724; doi:10.1371/journal.pone.0252434)
Supplement: S1 Appendix — (DOCX) [file pone.0252434.s001.docx]

**S1 Appendix. Database Search Strategy**

MEDLINE was searched using the Ovid Interface on 30/01/2020 for the period of 2001 to January 2020.

1. Abortion, Induced/ (39773)
2. Abortion, Legal/ (7457)
3. abortion.mp. (86470)
4. (Abortion, Induced/ OR Abortion, Legal/ OR abortion.mp) (87105)
5. Homeless Persons/ (7459)
6. Homeless Youth/ (1252)
7. homeless youth.mp (1463)
8. homeless adolescent*.mp (162)
9. homeless teen*.mp (14)
10. (Homeless Persons/ OR Homeless Youth/ homeless youth.mp OR homeless adolescent*.mp OR homeless teen*.mp) (8782)
11. Pregnancy/ (862987)
12. Pregnancy, Unplanned/ (1870)
13. Pregnancy, Unwanted/ (2587)
14. unwanted pregnancy.mp (1800)
15. unplanned pregnancy.mp (1280)
16. unintended pregnancy.mp (2676)
17. pregnancy.mp (946044)
18. Pregnancy/ OR Pregnancy, Unplanned/ OR Pregnancy, Unwanted/ OR unwanted pregnancy.mp OR unplanned pregnancy.mp OR unintended pregnancy OR pregnancy (946044)
19. 4 OR 18 (961140)
20. (4 or 14) AND 10 (207)

CINAHL was searched using the EBSCO Host interface on 30/01/2020 for the period of 2001 to January 2020.

1. Abortion, Induced (9441)
2. Attitude to Abortion (392)
3. abortion (17465)
4. (Abortion, Induced OR Attitude to Abortion OR abortion) (17465)
5. Homeless Persons (5107)
6. homeless youth (416)
7. homeless adolescent* (173)
8. homeless teen* (33)
9. (Homeless Persons OR homeless youth OR homeless adolescent* OR homeless teen*) (5387)
10. Pregnancy (181001)
11. Unwanted Pregnancy (1006)
12. Unplanned Pregnancy (2102)
13. unwanted pregnancy (1647)
14. unplanned pregnancy (2775)
15. unintended pregnancy (2784)
16. (Pregnancy OR Unwanted Pregnancy OR Unplanned Pregnancy or unwanted pregnancy or unplanned pregnancy or unintended pregnancy) (182536)
17. 4 OR 16 (189109)
18. 17 AND 9 (87)

EMBASE was searched directly on 30/01/2020 for the period of 2001 till January 2020.

1. “abortion”/de OR “unwanted pregnancy”/exp OR “unplanned pregnancy” (33,601)
2. abortion OR unplanned pregnancy OR unwanted pregnancy (127136)
3. 1 OR 2 (127136)
4. “homeless person” OR “homeless youth” (2181)
5. Homeless person or homeless youth or homeless adolescent* or homeless teen* (2212)
6. 4 OR 5 (2212)
7. 3 AND 6 (20)
